# Using the Machine Learning Method to Study the Environmental Footprints Embodied in Chinese Diet

**DOI:** 10.3390/ijerph17197349

**Published:** 2020-10-08

**Authors:** Yi Liang, Aixi Han, Li Chai, Hong Zhi

**Affiliations:** 1 College of Science, China Agricultural University, Beijing 100083, China; Cedricly1736@gmail.com; 2 International College Beijing, China Agricultural University, Beijing 100083, China; 2017314060118@cau.edu.cn (A.H.); luciazhi@163.com (H.Z.); 3 Chinese-Israeli International Center for Research and Training in Agriculture, China Agricultural University, Beijing 100083, China

**Keywords:** CHNS, water footprint, carbon footprint, ecological footprint, diet, machine learning

## Abstract

The food system profoundly affects the sustainable development of the environment and resources. Numerous studies have shown that the food consumption patterns of Chinese residents will bring certain pressure to the environment. Food consumption patterns have individual differences. Therefore, reducing the pressure of food consumption patterns on the environment requires the precise positioning of people with high consumption tendencies. Based on the related concepts of the machine learning method, this paper designs an identification method of the population with a high environmental footprint by using a decision tree as the core and realizes the automatic identification of a large number of users. By using the microdata provided by CHNS(the China Health and Nutrition Survey), we study the relationship between residents’ dietary intake and environmental resource consumption. First, we find that the impact of residents’ food system on the environment shows a certain logistic normal distribution trend. Then, through the decision tree algorithm, we find that four demographic characteristics of gender, income level, education level, and region have the greatest impact on residents’ environmental footprint, where the consumption trends of different characteristics are also significantly different. At the same time, we also use the decision tree to identify the population characteristics with high consumption tendency. This method can effectively improve the identification coverage and accuracy rate and promotes the improvement of residents’ food consumption patterns.

## 1. Introduction

Food, energy, and water (FEW) are the basic resources which are indispensable to human survival and social and economic development [1,2]. It is estimated that by 2030, the global demand for FEW will increase by 35%, 50%, and 40%, respectively [3]. At the same time, global issues such as food security, shortage of freshwater resources, and exhaustion of fossil energy are increasingly becoming "shortcomings" that restrict the development of modern society, seriously threatening national security and social stability. Moreover, there are intricate interactions (which we call the nexus) between FEW, and the nexus will be extremely significant for the sustainable development of future society [4]. As an important part of FEW, food consumption is an important factor affecting the environment. On the one hand, the food system is an important source of nutrients and energy for the human body. On the other hand, the production of food consumes environmental resources and can place great pressure on the environment. 

The 21st century has witnessed rapid changes in China’s society. With the progress of agriculture and the improvement of people’s living standards, Chinese people’s dietary demands have changed from focusing on the satisfaction of quantity to that of quality. Therefore, the food consumption structure is also undergoing a major change. Residents changed from consuming mainly plant-based foods to including more meat in their diet. From 1978 to 2012, the pork consumption of Chinese residents increased by four times, and beef consumption increased by 16 times [5]. Compared with plant foods, meat production emits more carbon, consumes more water resources, and occupies more land for every kilocalorie. For water resources, agriculture uses more freshwater than any other human activity, and the livestock industry needs to consume nearly a third of freshwater (most of which is used for feed cultivation) [6]. For example, the water consumption of beef production is 6.75 times that of the same quality cereal [7]. Meat production is an important source of methane, and its global warming potential is 21 times that of carbon dioxide [8]. In general, to ensure the sufficient production of grain, China has used a large amount of nitrogen-containing fertilizers in the past three decades, resulting in a nearly 345% increase in the carbon footprint [9]. At the same time, the food system, especially meat production, is an important source of nitrogen, phosphorus, and other pollutants, especially through the conversion of land into pasture and arable land forage crops, affecting biodiversity. Therefore, eating is not only a biological behavior but also an ecological behavior which has a great impact on the environment [10].

When evaluating the impact of the food system on the environment, water footprint, carbon footprint, and ecological footprint have become the most commonly used assessment indicators [11,12]. Based on the environmental footprint theory to analyze the resource and environmental impact of food consumption, and to discuss the ecological environmental load brought about by the transformation of food consumption, we can fully determine the consumption of water and cultivated land and other resources by consumption changes in a region. The resource guarantee required for food production is reasonably evaluated, so it has important practical significance [13].

The impact of the food system on the environment must be controlled within environmental tolerance. Improving the dietary structure of residents is undoubtedly one of the most effective methods. As to food producers and consumers, every aspect of the food life cycle can strive to reduce the impact of food on the environment [14]. We can advocate a balanced diet structure, reduce food waste, and cut down those unnecessary energy intakes [15,16,17]. Today, the global population’s food intake accounts for 19–29% of the total greenhouse gas emissions, 70% of freshwater consumption, and 38% land use [18,19]. By 2050, the world’s population will reach 9.7 billion people, which means that the dependence of the food system on the environment will undoubtedly further increase from current levels [20]. In addition, some scholars pointed out that climate change and the continuous urbanization process pose a huge challenge for the food supply [21,22]. Therefore, reasonable planning of food consumption to minimize the impact of the food system on the environment is conducive to achieving environmental sustainability [17,23,24].

A large number of scholars have carried out relevant research on the environmental footprint of the food system, mainly focusing on quantitative analysis, influencing factor analysis, and including multiple research scales such as global, national, and regional [25,26,27,28]. The Lancet once proposed a plan for the Planetary Health Diet, suggesting that we protect the environment and improve human health by reducing the production and consumption of red meat [27]. Based on the relevant data of DBI-16 (Diet Balance Index 2016 Version), Song et al. compared the different impacts of the food system on the environment under normal scenarios and diet optimization models, thus emphasizing the importance of improving diets in protecting the environment [28]. We can see that most researchers use the input–output model to conduct trade assessment, which is a top-down approach based on macro data to calculate the life cycle of resource consumption. Alternatively, by calculating the environmental footprint of different dietary structures, we can reveal the environmental benefits brought about by improving the dietary structure. This is actually a top-down method. Although it has a certain generality and has general guiding significance for the direction of policies and rules, it has certain defects in the individual accuracy of the sample. In fact, for policymakers, understanding the environmental benefits brought about by improved dietary structure can help them to better understand the importance and necessity of the food system, thus speeding up the advancement of related policies. After this, understanding the population characteristics of the environmental consumption of the food system can help them to identify people who are likely to require higher consumption, so as to accurately locate the implementation goals of related policies, which, therefore, has practical significance [13].

Machine learning is a multi-disciplinary cross-specialty, covering knowledge of probability theory, statistics, approximate theory, and knowledge of complex algorithms [29,30]. Computers are usually used as tools and committed to the real-time simulation of human learning methods, and existing content is divided into knowledge structures to effectively improve learning efficiency. Combined with machine learning, the simulation analysis of micro-statistical samples can more accurately identify the individual characteristics of the samples, with a certain degree of scientificity and accuracy. The China Health and Nutrition Survey (CHNS), focusing on micro-survey statistics, provided direct analysis from the perspective of subjects such as nutritional health science or environmental energy. In addition, it can also be combined with the database to expand the cross-section from an interdisciplinary perspective [31,32,33,34,35]. Therefore, the use of machine learning to filter and sort out the sample data in the CHNS database can help us to identify the characteristics of the population covered by the food system environment, thereby providing support for the implementation of related policies. The main contributions of this research are as follows: (1) summarize the overall distribution of water footprint, carbon footprint, and ecological footprint of Chinese residents’ food consumption; (2) examine the demographic factors which significantly affect the environmental footprint of people’s diets; (3) identify the group which has the highest environmental footprint in their diet. We hope that our research can bring certain guiding significance to environmental policy.

## 2. Materials and Methods 

**Figure 1:**
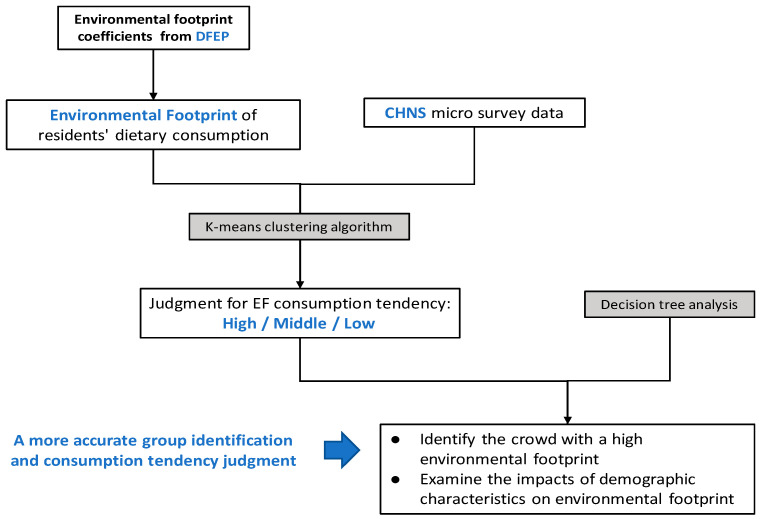
Research framework. Double Food–Environmental Pyramid (DFEP) provides the environmental footprint database; CHNS refers to the China Health and Nutrition Survey.

In order to identify the crowd whose diet has a high environmental footprint and examine how the demographic characteristics affect the environmental footprint, we combined machine learning methods such as K-means classification and decision trees to process the CHNS data. The framework of this research is shown in Figure 1:

### 2.1. Data Processing 

#### 2.1.1. Environmental Footprint Calculation

This study uses the CHNS data of 2011. The China Health and Nutrition Survey (CHNS) is an international survey project that has been ongoing for many years [36]. From the perspectives of different disciplines such as nutrition, economics, sociology, and demography, it investigates the effectiveness of the national implementation of nutrition and health policies while understanding the relationship between China’s economic transformation and residents’ healthy nutrition. CHNS uses random clustering and other statistical survey methods to select nearly 20,000 family members from more than 4000 families in different provinces among China. The surveyed people significantly vary in demographic characteristics. This provides space for more research related to people’s characteristics.

For the processing of CHNS data, first, we deleted the invalid survey samples, irrelevant variables, and irrelevant samples in the survey through data cleaning. Finally, we obtained 10,612 valid samples. These data contained the residents’ food consumption and their individual characteristics such as education, age, gender, income, etc. Combining the food consumption data of each sample from CHNS and the environmental footprint consumption per unit of this certain kind of food, the environmental footprint of each sample consumed in the food system can be calculated. The environmental footprint consumed by each sample can be calculated using the following formula:

(1) $$WF_{j}=\sum food_{i}\times wc_{i}$$

(2) $$CF_{j}=\sum food_{i}\times cc_{i}$$

(3) $$EF_{j}=\sum food_{i}\times ec_{i}$$

where $WF_{j}$ is the water footprint of the sample j, $CF_{j}$ is the carbon footprint of the sample j, and $EF_{j}$ is the ecological footprint of the sample j; $food_{i}$ is the amount of food *i* ingested by the sample j during the investigation period; $cc_{i}$, $wc_{i}$, and $ec_{i}$ refer to the coefficients for the water footprint, carbon footprint, and ecological footprint, respectively. The environmental footprint coefficients were obtained from the Double Food–Environmental Pyramid (DFEP) database [37].

Before the calculation, the units of every kind of food were adjusted to the same unit. The sum of various food consumption is the total environmental resource consumption of the corresponding sample. Then, combining the data of residents’ resource consumption and the previous operation results of the model, the significant outliers were screened to form the final dataset. 

#### 2.1.2. Cluster Analysis of Environmental Footprint Data

In order to distinguish the individual characteristics of environmental footprint data, we performed K-means clustering on the calculated water footprint, carbon footprint, and ecological footprint data. In order to correctly identify the three endogenous causes of environmental footprint consumption, we first divided each footprint dataset into three groups, high, medium, and low, which represent the three consumption levels for each environmental footprint. The K-means clustering algorithm is an iterative solution clustering analysis algorithm. For each kind of environmental footprint data, we first divided the dataset into 3 groups (high, medium, and low), and we randomly selected 3 objects as the initial cluster centers. Then, by calculating the distance between each object and each seed cluster center, each object was assigned to the cluster center closest to it to obtain three types of datasets, high, medium, and low, which also had some original features from the data.

Because the clustering data did not divide the number of samples into three groups on average, the number of samples in the high, middle, and low groups was significantly different. In order to balance the impact of sample number imbalance on machine learning training, this study used a randomly drawn imbalanced dataset. Since the number of data samples in each group was less than 6000, we used the random sampling method to repeatedly sample individuals from the original data group, so that the three data groups of high, medium, and low under each environmental footprint had 6000 samples; that is, each environmental footprint dataset was randomly selected and expanded into a 18,000 sample dataset. We used machine learning to analyze the driving factors of environmental footprint consumption.

Classification and regression are the two basic tasks of model building in machine learning. Among many machine learning algorithms, decision trees are more suitable for predicting categorical variables where the tree-like classification process is easy to present and understand; support vector machines, etc., involve more complicated mathematical algebra knowledge due to the widespread universality and reliability as a basic algorithm in data science. This study aims to analyze the driving factors of the environmental footprints of individual diets from micro statistics and classify individual residents’ footprints according to these driving factors. The extraction of classification labels can quickly rely on crowd characteristics to identify the crowd’s footprint consumption tendency. In the classification process, this study mainly used a decision tree classifier to classify the samples, but in the research process, the classification algorithm of support vector machine and the random forest was also used to test the decision tree classification results, which will be mentioned in the method comparison.

### 2.2. Applying Machine Learning Methods to the Environmental Footprint Data of Food Systems

**Figure 2:**
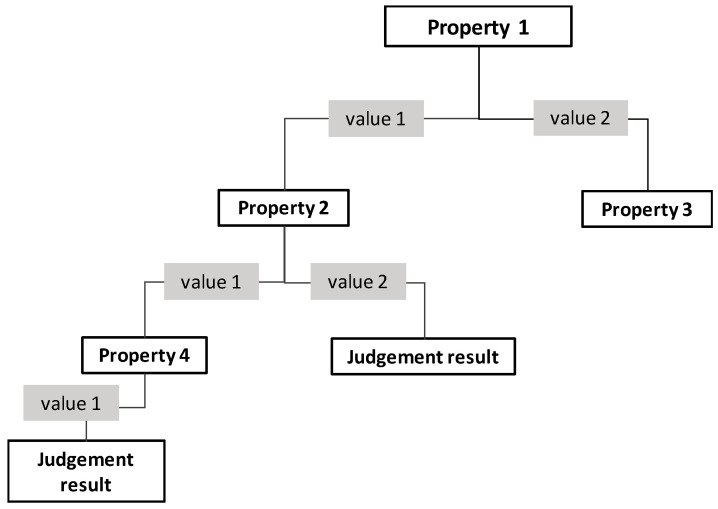
Schematic diagram of a decision tree.

The decision tree is a supervised machine learning method. Its basic idea is to classify samples layer by layer by selecting feature attributes and realize an agent based on feature judgment for data classification, feature selection, and other scenarios [38]. As shown in Figure 2, the decision tree algorithm will divide the samples layer by layer according to their attribute values and obtain the judgment results under different attribute combinations, thus forming a tree structure. Among them, the selected attribute is called the branch node, and the final judgment is called the leaf node. The node of the upper level in the branch node is called the root node of the next level.

**Figure 3:**
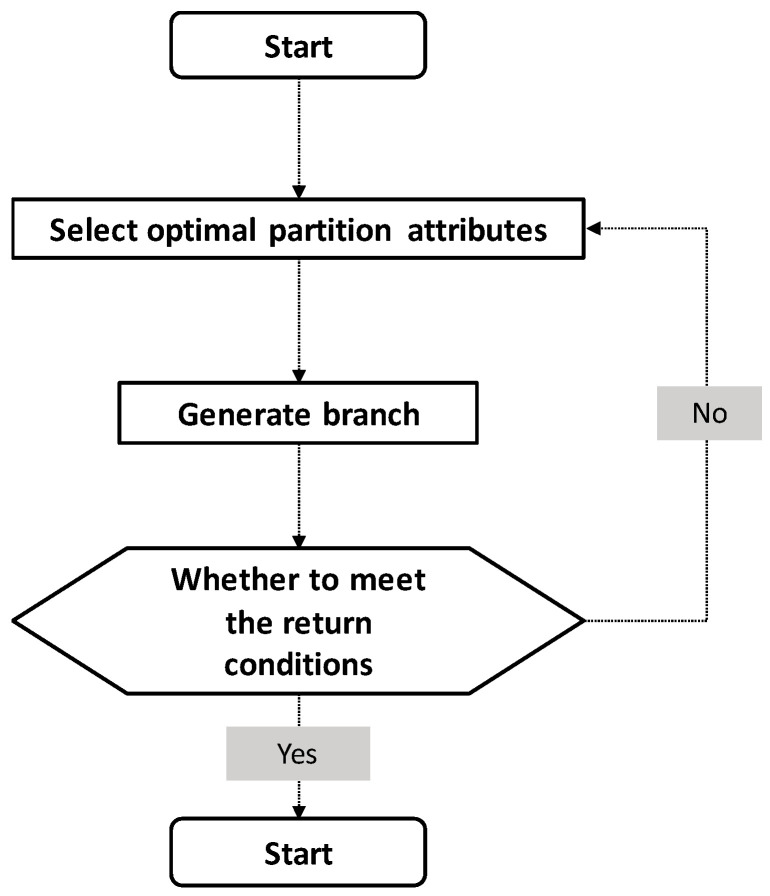
ID3 decision tree algorithm training steps.

The ID3 decision tree algorithm introduced in this article was proposed at the end of the last century and is the longest and most widely used decision tree algorithm [39]. ID3 introduces the concept of information entropy, which uses the gain to gain ratio of entropy as the decision criterion and selects toward the direction of greater information gain when making decisions, so as to better reduce the uncertainty of the sample and achieve a better classification effect. In our study, only one group of individuals was divided into two groups according to the entropy value, and the entropy value was calculated using a logarithm of 2 (other bases can be used). In the model, the original data group was divided into two groups according to the classification basis, and the two sets of entropy values were calculated separately. The classification standard with the lowest entropy value after data group classification is the optimal classification basis. The algorithm framework is shown in Figure 3, including three main implementation steps [40,41].

#### 2.2.1. Select the Optimal Partition Attribute

By defining information entropy, the ID3 decision tree selects information entropy as the basis for selecting the optimal partition attributes. Information entropy refers to the set purity of sample classification. The smaller the value of information entropy, the higher the purity of the sample set, and therefore, the higher the purity of the sample set after classification according to a certain attribute in the decision tree generation, which means a higher execution efficiency of the decision tree. The calculation formula of information entropy is as follows:

$$H\left(x\right)=-\underset{x}{\sum }p\left(x\right)\;log_{2}\left[p\left(x\right)\right]$$

where H(x) is the information entropy index of sample set x; p(x) is the proportion of a sample in this sample set. It can be seen from the above that the optimal partition attribute should be the attribute with the largest information entropy gain, namely:

$$a_{*}=\underset{a\in A}{\mathrm{argmax}}Gain\left(x,a\right)=\underset{a\in A}{\mathrm{argmax}}\left[H\left(x\right)-\overset{Va}{\underset{v=1}{\sum }}\frac{\left|x^{v}\right|}{\left|x\right|}H\left(x^{v}\right)\right]$$

where $a_{*}$ is the optimal partition attribute; Gain(x,a) represents the information entropy gain corresponding to the sample set x (information divergence) divided according to the attribute a; Va is the number of possible values of the attribute; $\left|x\right|\;\mathrm{and}\;\left|x^{v}\right|$ respectively represent the number of samples in the sample set *x* and the number of samples in the subset of samples whose attribute a is v in the sample set x; $H\left(x^{v}\right)$ is the information entropy of the sample subset.

#### 2.2.2. Generate Branches

For the selected optimal partition attribute, the original sample set is divided according to its possible values, and the number of attribute values is the number of branches.

#### 2.2.3. Determine Whether to Return

As shown in Figure 3, the decision tree generation process is a typical recursive problem decision-making process, and its return needs to meet one of the following three conditions: a. the sample subsets obtained after division are all of the same type and do not require further division; b. the optimal division attribute is empty set; c. the sample subset is an empty set.

## 3. Results

### 3.1. Normal Distribution of Environmental Footprint Consumption

**Figure 4:**
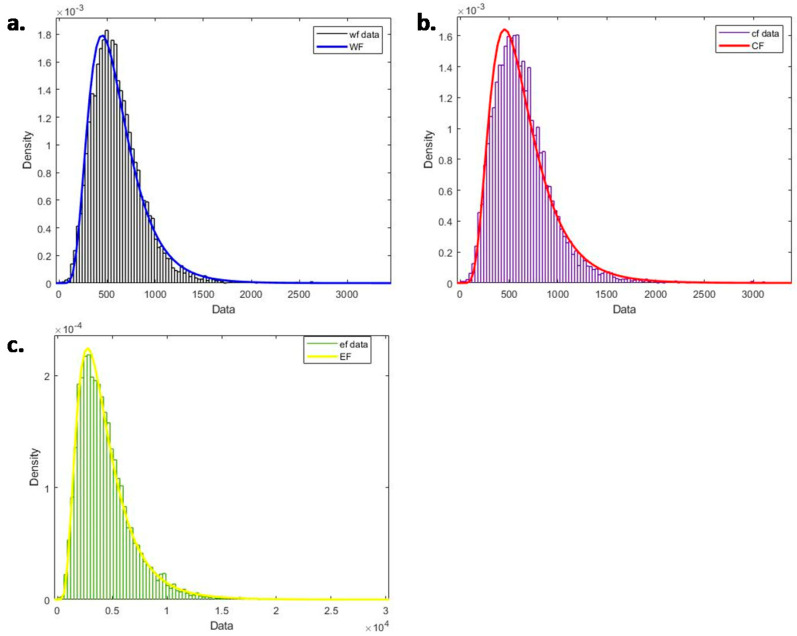
Distribution bar charts for environmental footprint consumption where (**a**) shows water footprint, (**b**) shows carbon footprint, and (**c**) shows ecological footprint.

By combining the environmental footprint data with the CHNS dataset, we finally calculated the environmental footprint of the food system of each sample. As shown in Figure 4, the environmental footprint consumption of the 10,612 samples was formed into a distribution bar chart.

For the water footprint, its central axis is around 500, which means that the consumption of water resources of the food system for most people (with a density of 1.8 × 10^−3^) is concentrated around 500 m^3^. For the carbon footprint, its central axis is around 500, which indicates that the emission of carbon dioxide of the food system is concentrated at around 500 kgCO_2_eq for most people (with a density of 1.6 × 10^−3^). For the ecological footprint, its central axis is around 2500, which means that the consumption of land resources of the food system for most people (with a density of 2.2 × 10^−4^) is concentrated at around 2500 gm^2^. In fact, it can be seen from Figure 4 that the consumption of the ecological footprint is more concentrated, while the consumption of the water footprint and carbon footprint is relatively more dispersed, especially the consumption of the carbon footprint. At the same time, judging from the ups and downs of the curve, the curves of the three environmental footprints are relatively different, showing significant individual differences. Overall, from Figure 4, we can see that the environmental footprint of the food system shows a tendency of skewness distribution, and the environmental footprint consumed by most people in this survey is less than average, with only a small percentage that is above average. At the same time, there are certainly individual differences in the food consumption of residents, and the environmental footprint of food consumption for the different samples is not evenly consumed, and the difference can even reach as much as three times.

From the data, we can see that, overall, the environmental footprint of the food system of most of our residents is at a low level, but there are also individuals with higher consumption. This result is consistent with the results of the food and food consumption distribution research of the United Nations Food and Agriculture Organization [42]. It can be seen that the environmental footprint of the food system is fundamentally determined by the food consumption pattern. Everybody needs a healthy diet, and the food system should provide enough food supply to guarantee their health. However, if the amount of food consumption exceeds the amount that the human body needs by too much, this will harm the human body. At the same time, the excess food can be regarded as a kind of waste of resources. García-Herrero et al. pointed out that reducing food loss is considered to be an important means to improve food security and reduce the pressure on natural resources [43]. From the CHNS dataset, there is no doubt that the group of people who have more than 1500 m^3^ consumes more than what they need when compared to most people in the survey. Therefore, we can conclude that, while some people may have been confronting the threat of hunger, other people create unnecessary food waste at the same time, contributing to the imbalanced consumption of their environmental footprint.

### 3.2. Identify Population Characteristics of Environmental Footprint in Food System

If the food system can be improved and transfer food from people with over-consumption to under-intake, this will be a good way to improve the status quo of food consumption and reduce waste and the environmental footprint. If we know which population has a higher tendency to consume more food and advise them to consume less food according to their individual characteristics, this should be more effective than an exhortation to the public. Therefore, we used the decision tree to judge the individual characteristics of the environmental footprint of the food system.

Unlike top-down macro-data research, this study focuses more on individual characteristics to separate the higher from the lower based on direct consumption calculated with micro-data (derived from CHNS). From the number of samples in the two groups, it can be concluded that the male to female sex ratio in the CHNS dataset is not 1:1 but is close to 1.1:1. At the same time, the data show that the number of low income and low education groups is much higher than that of high income and high education groups, which is the basic feature of the survey data from CHNS and is also in line with the actual population ratio. By using the decision tree classifier, we ranked the main factors affecting the three environmental footprints separately. The main basis for ranking is the magnitude of its entropy decrease relative to 1.585 (related to log 2). It is an ideal state that, after a split, the entropy of one set returns to 0. Therefore, for each split, the greater reduction is better as it means that it is closer to the ideal situation. The larger the degree of decline, the greater its influence, so it ranks higher. The resulting order is as follows.

By using the decision tree classifier to classify the characteristics of the CHNS samples, the three most distinguishable characteristics were selected due to entropy by the classifier: water footprint (gender, income level, and education level); carbon footprint (gender, education, and income level); ecological footprint (income, region, and education level). It was noted that for income level, we divided the sample into two groups: low-income level and high-income level. In addition, because the sample variables are similar, we further subdivided them on this basis to better identify the footprint consumption tendency in the next step. For the low-income group, we divided it into very low, low, and middle income, and for the high-income group, we divided it into high and very high income. Our correlation test also confirms this classification. We used the Pearson test to analyze the correlation of 7 demographic characteristics. Among them, BMI and physical activity have a strong correlation with other items and should not be considered as independent factors. As a result, the five basic demographic indicators, which are age, gender, region, income level, and education level, have shown an insignificant correlation with others and should be regarded as independent factors for further analysis (shown in supplementary material as an excel).

For the water footprint, we can see in Table 1 that gender can be regarded as the most distinguishable characteristic—that is, the most influential factor. We can see that the entropy value for gender decreased from 1.585 to 1.556 for males and 1.553 for females. Then comes the income level, where the entropy value decreased from 1.585 to 1.573 for the low-income group and 1.564 for the high-income group. The third factor is education level. Obviously, after only one classification, the data entropy of male and female had a limited decrease, but it was still the most influential index among various indicators, which shows that after classification, the tendency to distinguish consumption of resources and environment already occurred in the male or female data group.

For the carbon footprint, gender was also the most distinguishable characteristic, and the entropy value decreased from 1.585 to 1.548 for males and 1.54 for females (Table 2). Here, in the model, the dataset of carbon footprint which uses gender as the root node has a better reduction of entropy than the income level or the education level. However, as the entropy of male (female) shows, the male set and the female set both consist of samples that can still be split. There is no difficulty to understand the result that, in the male or female set, samples consist of high, middle, and low cases, for there are plenty of people who consume less food no matter what their gender is. It is common sense that males need slightly more than females for their basal metabolism, which leads to a higher tendency to consume more and a higher value of all three kinds of footprints shown in the next result section. For the classification index of education level, the entropy value decreased from 1.585 before classification to 1.573 for the low education level group and 1.559 for the high education level group. As for the income level, it fell to 1.577 in the low-income group and 1.57 in the high-income group after classification.

Unlike WF and CF, as for ecological footprint, the most distinguishable factor is income level. The entropy value decreased from 1.585 to 1.539 for the low-income group and 1.528 for the high-income group (Table 3). The second factor is the region and the third is the education level. In general, there are four indicators that have the greatest impact on these three footprints, namely gender, education level, income level, and region, where the classification index of the region only appears in the ecological footprint.

### 3.3. Comparison of the Consumption Ranking of Various Types of People under the Main Index Classification

Judging the purity of the sample set obtained after classification according to a certain attribute through information entropy can help us to identify the influencing factors of the environmental footprint of the food system. This is a forward-thinking process from root to leaf. Conversely, if we reverse the process from the leaf to the root, we can obtain the characteristics of the three types of environmental footprint of the high-consuming groups, and then we can take relevant measures for high-consuming groups. Through the K-means clustering method, three environmental footprint consumption tendencies can be divided into high, medium, and low. Combining the results with the three-level feature analysis performed by the decision tree (eight categories can finally be obtained), we can obtain the environmental footprint consumption tendency judgments of the eight categories. Finally, based on the percentage, we can obtain the judgment of the environmental footprint consumption tendency of this category of people.

#### 3.3.1. The Characteristics of Water Footprint High-Consuming Groups

**Figure 5:**
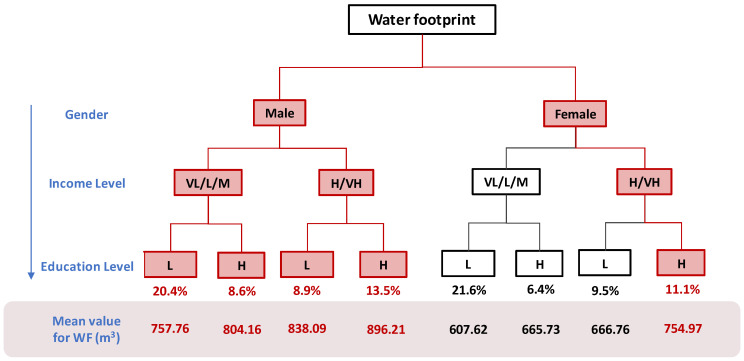
A decision tree for judgment of the water footprint consumption tendency where the red color indicates a high consumption tendency. The percentage number under the decision tree represents the percentage of the number of samples under the category to the total number of samples. The real number indicates the average water footprint of 8 groups of people (m^3^ per year). For income level, VL, L and M mean very low, low and middle income level respectively, while H and VH mean high and very high income level. For education level, L and H mean low and high education level respectively.

The relevant results are input into the decision tree diagram in Figure 5. The red part of the diagram represents the characteristics of people who tend to demonstrate a high water footprint. We can find that a total of 62.5% of the population tends to demonstrate a high water footprint. Not only does this percentage exceed 50%, but it is also the largest compared to the other two footprints, indicating that, overall, more than half of the population also shows a higher propensity to consume water. Therefore, the consumption tendency for the water footprint should be the main focus. As shown in Figure 5, the water footprints of people with high consumption propensity are 757.76 m^3^ (for males in the low-income group with low education level), 804.16 m^3^ (for males in the low-income group with high education level), 838.09 m^3^ (for males in the high-income group with low education level), 896.21 m^3^ (for males in the high-income group with high education level), and 754.97 m^3^ (for females in the high-income group with high education level). The average water footprint demonstrated by the eight groups of people is 749 m^3^, but the average water footprint of the five high-consumption propensity populations is 810 m^3^, which is 8% higher than the average. At the same time, we can find that males in the high-income group with high education level have the highest water footprint. This part of the population accounts for a larger percentage of the overall population, which is 13.5%, so this type of population needs to be focused on.

#### 3.3.2. The Characteristics of Carbon Footprint High-Consuming Groups

**Figure 6:**
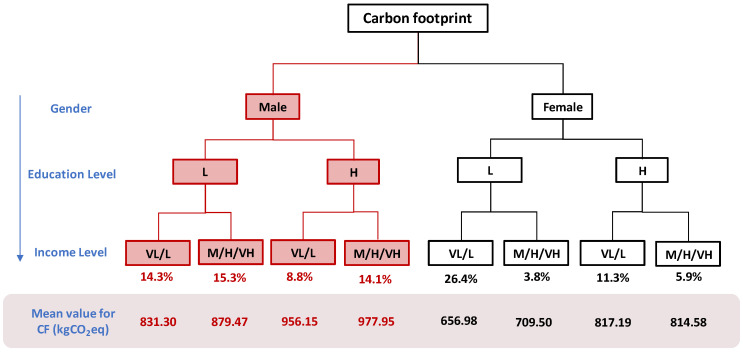
A decision tree for judgment of the carbon footprint consumption tendency where the red color indicates a high consumption tendency. The percentage under the decision tree represents the percentage of the number of samples under the category to the total number of samples. The real number indicates the average carbon footprint of 8 groups of people (kgCO_2_eq per year). For income level, VL, L and M mean very low, low and middle income level respectively, while H and VH mean high and very high income level. For education level, L and H mean low and high education level respectively.

Regarding the carbon footprint, the data shows a more pronounced gender difference. For males, regardless of the difference in income level or education level, the proportion of people with high carbon footprint and propensity to consume is relatively large. The female group generally has a lower carbon footprint and consumption tendency. Entering the relevant results into a decision tree diagram, as shown in Figure 6, we can clearly see the gender difference in the carbon footprint of the food system. People with high consumption tendency account for 52.5% of the total, which means that more than half of the population will show a high consumption tendency in their carbon footprint.

From Figure 6, we can find that the carbon footprints of people with high consumption propensity are 831.30 kgCO_2_eq (for males in low-income groups with low education level), 879.47 kgCO_2_eq (for males in high-income groups with low education level), 956.15 kgCO_2_eq (for males in low-income groups with high education level), and 977.95 kgCO_2_eq (for males in high-income groups with high education level). The average carbon footprint of the eight populations is 830 kgCO_2_eq, and the average carbon footprint of the four high-consumption propensity populations is 911 kgCO_2_eq, which is 10% higher than the average. At the same time, we can find that males in high-income groups with high education levels have the highest carbon footprint. This part of the population accounts for a larger percentage of the total, 14.1%. Therefore, this type of population needs to be focused on.

#### 3.3.3. The Characteristics of Ecological Footprint High-Consuming Groups

**Figure 7:**
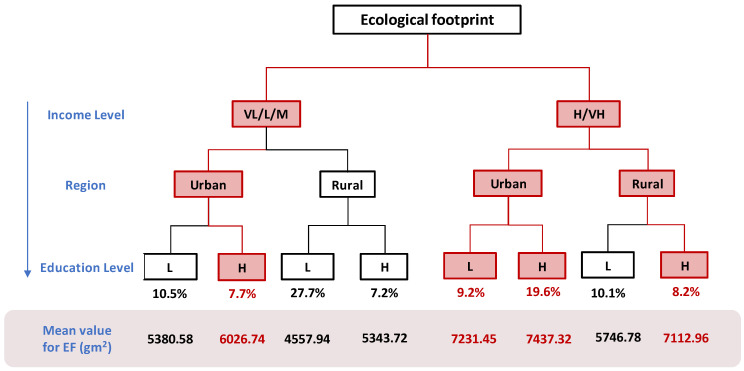
A decision tree for judgment of the ecological footprint consumption tendency where the red color indicates a high consumption tendency. The percentage under the decision tree represents the percentage of the number of samples under the category to the total number of samples. The real number indicates the average carbon footprint of 8 groups of people (gm^2^ per year). For income level, VL, L and M mean very low, low and middle income level respectively, while H and VH mean high and very high income level. For education level, L and H mean low and high education level respectively.

For the ecological footprint, the relationship between its consumption tendency and gender is weak. As shown in Figure 7, it can be found that there are four types of people showing a high consumption trend, namely:

- low-income groups with high education levels who live in urban areas;
- high-income groups with low education levels who live in urban areas;
- high-income groups with high education levels who live in urban areas;
- high-income groups with high education levels who live in rural areas.

In summary, high-income groups or people with high education levels generally have a higher tendency to generate an ecological footprint. The population with high consumption tendency accounts for 44.7% of the overall population, which means that less than half of the population will show a high consumption tendency in their ecological footprint.

It can be seen from Figure 7 that the ecological footprints of people with high consumption propensity are 6026.74 gm^2^ (for low-income groups with higher education levels who live in urban areas) and 7231.45 gm^2^ (for high-income groups with lower education levels who live in urban areas), 7437.32 gm^2^ (for high-income groups with higher education levels who live in urban areas), and 7112.96 gm^2^ (for high-income groups with higher education levels who live in rural areas). The ecological footprint of the average consumption of eight groups of people is 6105 gm^2^. Among them, the average ecological footprint of these four high-consumption-prone groups is 6952 gm^2^, which is 14% higher than the average. At the same time, we can find that high-income groups with higher education levels who live in urban areas generate the highest ecological footprint. It is worth noting that this part of the population accounts for a larger percentage of the overall, 19.6%, so this category of people needs to be focused on.

## 4. Discussion

### 4.1. Comparison with Previous Research and Implications of This Study 

In the classification of water resource consumption, carbon emissions, and ecological footprint consumption tendency, we used a decision tree classifier to independently classify the three types of consumption, but the final classification results have different degrees of similarity. 

Ulucak et al. explored the impact of this demographic feature on the environment by studying the relationship between national income levels and environmental footprint [44]. They found that the ecological footprint first tends to increase at the initial income level and then gradually decreases through the economic growth of each income group in the country. On this basis, trade liberalization has led to an increase in the ecological footprint, while human capital has led to a decline in the environmental footprints of countries in each income group. At the same time, Fang, Chen, and others believe that the level of education plays a vital role in environmental issues [45]. For Chankrajang and Muttarak, they believe that by improving the acquisition of knowledge, values, and priorities, as well as the ability to plan for the future and the efficiency of resource allocation, education can enable people to better understand climate change and other complex environmental information [46]. Elena and others analyzed the impact of regions on the environmental footprint and found that in the food system, urban consumption is higher than that in rural areas [47].

In view of the current situation in China, we found that the common characteristics of the three groups with high propensity to generate environmental footprints are high income level and high education level. First of all, it can be concluded that China is currently undergoing rapid development. As economic income continues to increase, high-income groups tend to consume more food. At the same time, due to the country’s overall lack of awareness of environmental protection and the lack of awareness of the environmental costs of the food system, the high-income population tends to consume more refined foods, which leads to a higher tendency to generate environmental footprints [48,49]. Regarding the level of education, our data show that people with high levels of education may generate greater environmental footprints in the food system, which is also caused by the lack of awareness of environmental protection in the country as a whole. We speculate that China’s education system lacks explanation and analysis of the concept of environmental protection. At the same time, highly educated people may better understand the value of food, which may lead to increased intake of high-resource foods (such as eggs and milk). For the ecological footprint, we can see that region (urban versus rural) is a more important influencing factor, while gender is relatively weak. This is due to the uneven distribution of resources between urban and rural areas, and people are paying different levels of attention to the issue of waste recycling. For example, in rural areas, vegetable waste is reused as fertilizer, while urban people mix the waste with non-biodegradable waste.

At this stage, food is placing great pressure on the ecological environment. Through this classification, high propensity can reversely identify the characteristics of groups with high resource consumption, thereby providing an identification basis for directional reduction of environmental resource consumption caused by food consumption. At the same time, for high-consumption groups with different group characteristics, government departments can formulate corresponding propaganda programs according to the group characteristics. Regarding the resource and environmental consumption caused by residents’ food, compared with some studies that analyze the consumption of residents’ food footprints from the perspective of provinces and cities, this study analyzes from a national-level regional scope in China by combining three types of footprint [13,50]. The larger data area can better exclude the overall impact caused by the diet characteristics of the provinces, cities, and regions, and the analysis of the three footprints using the same dataset can make horizontal comparisons to find the characteristics of common residents that affect footprint consumption and our results of the study also confirm this idea well.

### 4.2. Strengths and Shortages 

The study found that residents’ daily water intake, water resources, carbon emissions, and environmental consumption showed a logarithmic normal distribution trend, and most residents’ consumption was relatively concentrated. At the same time, it was found that the three characteristics of residents, such as gender, income level, and low residence, had the most significant impact on residents’ resource consumption. Among them, people with different characteristics also had different resource consumption trends. According to the consumption tendency, the population of different special recruits is targeted, and the dietary habits are changed to reduce the pressure on the resource environment. In this way, the efficiency of the targeted change is unmatched by the popularity. In addition, this study also combined the microscopic data of residents’ dietary intake with machine learning, as well as the discipline of environmental ecology, which promoted the ability to process the data of residents’ onlookers.

Random forest can be regarded as an integrated learning algorithm formed by multiple decision trees, while SVC (Support Vector Classification) realizes classification through mathematical algebraic transformation. Compared with the other two models, the decision tree focuses on providing an intuitive and easy-to-understand classification method for algorithm users. The decision tree has the strongest interpretability and visualization ability. The learning effect of random forest is usually due to a single decision tree model, and the integrated learning algorithm can independently process each tree model in the processor in the cluster, but the random forest does not have the good scalability and visualization performance of the decision tree. Support vector machine classification can use a variety of kernel functions to transform small sample data, but the strong mathematical theory support also leads to the lower interpretability of the model; the model is more difficult to understand, and the matrix search operation for selecting parameters in the model stores a large amount of data. When the sample participates in the calculation, it will consume a lot of calculation time and calculation performance, and the convergence and divergence of the model also depends on the adjustment parameters. However, the decision tree uses a greedy search strategy, which may lead to overfitting or high variance, which is better than SVM (Support Vector Classification). Compared with other commonly used factor recognition algorithms, such as regression fitting or LASSO dimensionality reduction, etc., the decision tree can analyze a variety of factors to obtain the degree of factor contribution under different scenarios.

While this study proposes a classification to distinguish the water resource consumption, carbon emissions and ecological land use of the population, there are also some research limitations. First of all, the number of samples of the population surveyed in the CHNS survey report is relatively small, and the sample distribution does not completely cover all provinces and cities in China, which may result in the sampling of samples being unrepresentative. When investigating various food intakes, individual incomes, and other data of sample individuals, it is difficult to ensure that the sampled individuals report the statistics of their intake truthfully. The reasons may be that individuals forget to report due to negligence, and individuals may underreport in order to increase privacy; individuals may refuse to investigate and report for other reasons. The official data of the CHNS survey are currently only updated to 2015. Among them, the latest data released in the food survey related to 2011. There is still a certain time gap from today, which may lead to lag in the model and results. Secondly, because the classification of individual residents’ natural resource consumption tendency is only based on cluster analysis at the data processing level, it does not further combine the scientific significance of resource consumption in resource and environmental disciplines and has certain limitations. The following research can be further improved by further exploring the classification of resources and environmental consumption caused by residents’ food intake, in order to obtain more objective disciplinary classification results and at the same time give the results more disciplinary significance. Finally, this research considers only a combination of data processing algorithms, environmental ecology disciplines, and residents’ food consumption. The methods involved may be relatively simple methods in a single discipline, and they can be combined in different studies. Advanced analysis methods in the discipline direction could carry out more in-depth scientific research in interdisciplinary fields.

## 5. Conclusions

By studying the relationship between residents’ dietary intake and environmental resource consumption, we found that residents’ environmental footprints in the food system show a logistic normal distribution trend. At the same time, there are three population characteristics, (gender, income level, and education level) that have the greatest impact on this trend, where those demographic characteristics are also clearly distinguished. We determined the group characterized by a high consumption tendency in order to reduce the environmental resource consumption caused by food intake in a targeted manner. Targeted improvements and countermeasures will undoubtedly increase the efficiency of the structure and total improvement. By combining food science and environmental sustainability, we use data science to analyze relevant data and more accurately identify high-consumption groups with large environmental footprints. The machine learning method is used to identify the characteristics of the population, which can provide a basis for the accurate delivery of policies; this method is proven to be instructive. It is hoped that our research can bring people to an appropriate understanding of the environmental footprint of the food system, thereby promoting the realization of the sustainable development goals.

## Figures and Tables

**Table 1:** Top three distinguishable factors for water footprint consumption.

Rank	1st		2nd		3rd	
Influence Factor	Gender		Income Level		Education Level	
Classification	male	female	very low to middle	high to very high	low	high
Entropy Value	1.556	1.553	1.573	1.564	1.575	1.562
Information Divergence	0.029	0.032	0.012	0.021	0.01	0.023

**Table 2:** Top three distinguishable factors for carbon footprint consumption.

Rank	1st		2nd		3rd	
Influence Factor	Gender		Education Level		Income Level	
Classification	male	female	low	high	very low to middle	high to very high
Entropy Value	1.548	1.540	1.573	1.559	1.577	1.57
Information Divergence	0.037	0.045	0.012	0.026	0.008	0.015

**Table 3:** Top three distinguishable factors for ecological footprint consumption.

Rank	1st		2nd		3rd	
Influence Factor	Income Level		Region		Education Level	
Classification	very low to middle	high to very high	urban	rural	low	high
Entropy Value	1.539	1.528	1.545	1.553	1.56	1.541
Information Divergence	0.046	0.057	0.04	0.032	0.025	0.044

## Supplementary Materials

The following are available online at https://www.mdpi.com/1660-4601/17/19/7349/s1.
